# Temporal and spatial variability in the nutritive value of pasture vegetation and supplement feedstuffs for domestic ruminants in Western Kenya

**DOI:** 10.5713/ajas.18.0114

**Published:** 2018-07-26

**Authors:** Alice Anyango Onyango, Uta Dickhoefer, Mariana Cristina Rufino, Klaus Butterbach-Bahl, John Patrick Goopy

**Affiliations:** 1 Animal Nutrition and Rangeland Management in the Tropics and Subtropics, Institute of Agricultural Sciences in the Tropics (Hans-Ruthenberg Institute), University of Hohenheim, Fruwirthstr. 31, 70599 Stuttgart, Germany; 2 International Livestock Research Institute, P.O Box 30709-00100, Nairobi, Kenya; 3 Maseno University, Private Bag 40105, Maseno, Kenya; 4 Lancaster Environment Centre, Lancaster University, Lancaster LA1 4YQ, United Kingdom; 5 Karlsruhe Institute of Technology, Institute for Meteorology and Climate Research, Atmospheric Environmental Research, Kreuzeckbahnstr. 19, 82467 Garmisch-Partenkirchen, Germany

**Keywords:** Feed Evaluation, Grazing Livestock, Pasture Herbage, Ruminant Nutrition, Seasonal Variation

## Abstract

**Objective:**

The study aimed at quantifying seasonal and spatial variations in availability and nutritive value of herbaceous vegetation on native pastures and supplement feedstuffs for domestic ruminants in Western Kenya.

**Methods:**

Samples of herbaceous pasture vegetation (n = 75) and local supplement feedstuffs (n = 46) for cattle, sheep, and goats were collected in 20 villages of three geographic zones (Highlands, Mid-slopes, Lowlands) in Lower Nyando, Western Kenya, over four seasons of one year. Concentrations of dry matter (DM), crude ash (CA), ether extract (EE), crude protein (CP), neutral detergent fibre (NDF), gross energy (GE), and minerals were determined. Apparent total tract organic matter digestibility (dOM) was estimated from *in vitro* gas production and proximate nutrient concentrations or chemical composition alone using published prediction equations.

**Results:**

Nutrient, energy, and mineral concentrations were 52 to 168 g CA, 367 to 741 g NDF, 32 to 140 g CP, 6 to 45 g EE, 14.5 to 18.8 MJ GE, 7.0 to 54.2 g potassium, 0.01 to 0.47 g sodium, 136 to 1825 mg iron, and 0.07 to 0.52 mg selenium/kg DM. The dOM was 416 to 650 g/kg organic matter but differed depending on the estimation method. Nutritive value of pasture herbage was superior to most supplement feedstuffs, but its value strongly declined in the driest season. Biomass yields and concentrations of CP and potassium in pasture herbage were highest in the Highlands amongst the three zones.

**Conclusion:**

Availability and nutritive value of pasture herbage and supplement feedstuffs greatly vary between seasons and geographical zones, suggesting need for season- and region-specific feeding strategies. Local supplement feedstuffs partly compensate for nutritional deficiencies. However, equations to accurately predict dOM and improved knowledge on nutritional characteristics of tropical ruminant feedstuffs are needed to enhance livestock production in this and similar environments.

## INTRODUCTION

Ruminant production in sub-Saharan Africa largely depends on grazing native pastures and feeding of crop residues and agricultural by-products as dry-season supplements [1]. These crop residues and by-products tend to be rich in fibre and low in metabolizable energy (ME), crude protein (CP), and minerals, thereby limiting feed intake, diet digestibility [2], and performance of domestic ruminants.

Livestock production is an important sector in Kenyan economy where smallholder systems contribute three-quarters of total agricultural output [3]. Smallholder systems in Western Kenya have constraints in provision of sufficient nutritive value and quantity of feedstuffs throughout the year [4]. Moreover, mineral deficiencies are common in ruminants in the Rift Valley region [5] and commercial supplements are not always affordable to smallholder livestock farmers. Climatic and edaphic factors control primary production, species composition, and nutritive value of feedstuffs for grazing livestock [6] which may result in pronounced small-scale spatial and temporal differences in the yield and nutritional value of available feed resources and thus the need for region- and season-specific solutions to improve animal nutrition and performance.

Objectives of the study were therefore to quantify seasonal and spatial variations in the availability and nutritive value of tropical pasture herbage and supplement feedstuffs for grazing domestic ruminants in Western Kenya and to evaluate the need for local and season-specific solutions to improve livestock feeding. It was hypothesized that biomass yield (BY) and nutritive value of the pasture vegetation grazed by animals are highly variable between seasons and geographic zones; however, that local supplement feedstuffs are suitable to compensate for nutritional deficiencies in the pasture herbage and to develop region-specific solutions for improved livestock feeding and production.

## MATERIALS AND METHODS

### Study area

The study was conducted in a 100 km^2^ area (00°13′ S to 00°24′ S, 34°54′ E to 35°4′ E) in Lower Nyando Basin, Western Kenya. The site was selected to represent three distinct geographies common in the area, refered to as ‘zones’: Lowlands (0% to 12% gradient), Mid-slopes (12% to 47% gradient, steeper at the escarpment), and Highlands (>47% gradient at escarpments, 0% to 5% at the plateau) at altitudes of 1,200 to 1,750 m above sea level [7]. Soils of the Lowlands are sandy-clays to silty-loamy with visible effects of soil erosion and land degradation; the Mid-slopes are clay and silty loams, while the Highlands are silty to loamy. Two-fifths of the land cover is rangelands mainly used for grazing livestock [7]. Detailed description of the area is available in [8]. Mixed crop-livestock systems are predominant. Livestock consist of cattle, sheep, goats, chicken, and donkeys. The main cattle breeds are East African shorthorn zebus and zebu×*Bos taurus* in the commercial dairy-oriented Highlands.

**Figure 1:**
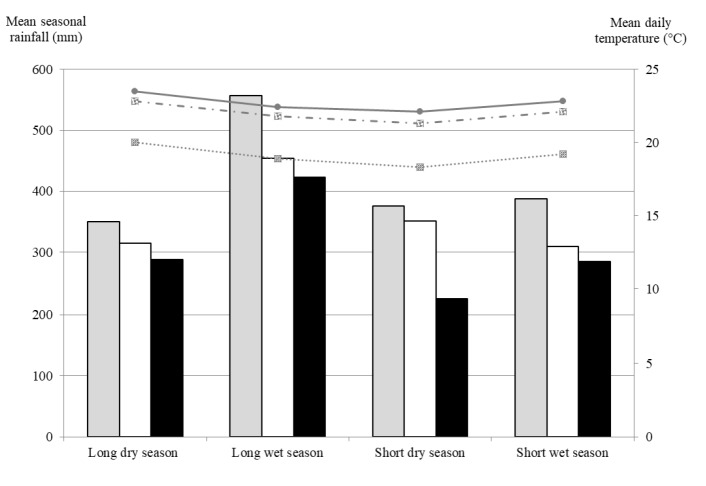
Mean seasonal rainfall and daily mean ambient air temperatures (1982 through 2012) for the three zones in Lower Nyando, Western Kenya. Source: Climate-data.org (http://en.climate-data.org).

The climate is humid to sub-humid. The annual rainfall is 1,200 to 1,725 mm with a bi-modal pattern allowing for two cropping seasons a year. The four climatic seasons are long dry season (January to March), long wet season (April to June), short dry season (July to September), and short wet season (October to December) (Figure 1). The first two climatic seasons fall in the long rainy cropping season, and the last two fall in the short rainy cropping season.

Based on results of an earlier survey conducted in the area using 200 households (IMPACTLite), 20 villages were selected (for details see [9]). Sample size of 60 households was based on a total population of 7,528 households, at 95% confidence level, 5% margin of error, and 10% variability. Proportional to size probability sampling with replacement based on clustering of the households in the IMPACTlite dataset yielded 24 farmers in the Lowlands, 18 in the Mid-slopes, and 18 in the Highlands [10].

### Sample collection and processing

Herbaceous pasture vegetation in the study area is predominantly composed of grasses such as *Digitaria gazensis* Rendle, *D. ciliaris* (Retz.) Koeler, *Eragrostis superba* Peyr., *E. aspera* (Jacq.) Nees, *Hyparrhenia collina* (Pigl.) Stapf, *Cynodon dactylon* (L.) Pers., *Cappillipedium parviflorum* (R. Br.) Stapf, and *Bracharia* spp. [7]. There are a few herbaceous dicots such as *Commelina africana* L., *Portulaca olearaceae* L., *Solanum incanum* L. 1753, and *Ipomea obscura* (L.) Ker Gawl [7]. Ligneous species were not included in the pasture vegetation, because the most abundant species were also collected either as mixed browsed leaves (MBL), or individually as outlined below. Above-ground BY of the herbaceous pasture vegetation was determined using enclosure cages to prevent livestock grazing and trampling. A wire mesh cage (0.5×0.5×0.5 m) was placed on the pasture of a randomly selected household per village, assuming the village pastures were homogenous. Hence, a total of eight cages were placed in the Lowlands, six in the Mid-slopes, and six in the Highlands. In August 2014, November 2014, February 2015, and May 2015 (i.e., coinciding with the middle of the four different seasons), the above-ground plant biomass within the cage was manually clipped at about 2.5 cm above the ground using a pair of scissors. All harvested plant material was packed into a pre-weighed paper bag, weighed (Citizen scale Model CTG6H, accuracy 0.1 g; Piscataway, NJ, USA), and the fresh weight recorded. Thereafter, the cage was placed back in the same location until the next sampling. A total of 75 samples of all the above-ground plant biomass material harvested were collected (i.e., 20 cages for each sampling less five tampered with by farmers or animals). Samples for each season were later pooled for all analyses on the basis of proximity of the villages to each other within the same zone (i.e., the Lowlands five samples, the Mid-slopes three samples, and the Highlands three samples) resulting in 44 pasture samples.

A total of 62 samples of supplement feedstuffs offered by farmers at the homestead (i.e., MBL, banana [*Musa* ssp.] leaves and pseudostem, sweet potato [*Ipomoea batatas*] vines [SPV], sugarcane tops [*Saccharum officinarum*], Napier grass [*Pennisetum purpureum*], swamp reeds [*Cyperus papyrus*], maize [*Zea mays*] thinnings, and rice [*Oryza sativa*] stover/husks) were collected in February 2014 (i.e., dry-season feedstuffs) and May 2015 (i.e., wet-season feedstuffs). Samples of the MBL (fed to cattle as ‘cut and carry’ during the dry season) mainly comprised leaves and twigs of *Lantana camara* L., *Terminalia brownie* Fresen, *Rhus natalensis* Bernh. ex Krauss, *Tithonia* spp., *Carissa edulis* Vahl, *Grewia bicolor* Juss., *Harrisonia abyssinica* Oliv., *Aphania senegalensis* (Juss. ex Poir.) Radlk., *Thevetia peruviana* (Pers.) K. Schum., *Vepris nobilis* (Delile) Mziray, *Combretum molle* R. Br. ex G. Don, *Senna siamea* Lam., *Acacia* spp., *Crotalaria* spp., *Gliricidia* spp., *Grevellia* spp., and *Citrus limon* (L.) Burm. f. among others identified based on farmers’ knowledge of common browsed species. At each household offering these leaves (mainly in the Mid-slopes and Lowlands), a twig of each from at least four trees or shrubs of each available species (about 30 cm long) was cut using a pair of scissors. The twigs were then cut into smaller pieces, pooled, and about 300 to 500 g of the sample were packed into a pre-weighed paper bag, weighed again (Citizen scale Model CTG6H, Piscataway, NJ, USA; accuracy 0.1 g), and the weight recorded. About 300 to 500 g of the leaves of *Mangifera indica* L. (MIL), and *Balanite aegyptiaca* (L.) Delile (BAL) were collected and analysed separately, because, according to farmer information and own observations, these tree species form a large part of the diets of ruminants, especially during the dry season. Banana leaves were collected following the normal practice used by farmers to harvest it (i.e., gathering the oldest green leaves). Leaves of four banana plants were cut where the leaf joins the petiole, chopped using a machete, pooled, and about 1 kg of the sample was packed into a pre-weighed paper bag, weighed again, and the weight recorded. About 20 cm length of banana pseudostem was cut from at least four freshly cut plant stumps. These were then treated in the same way as the banana leaves. Sugarcane tops, SPV, Napier grass, swamp reeds, maize thinnings (composed of thin weak maize plants pulled out when weeding of maize farms), and rice husks/stover were sampled from heaps already on offer to the animals by first homogenizing the material and taking a representative sample. Feedstuffs were collected from all the households that used such feedstuffs and later the samples were sorted such that the same feed type collected in a geographical zone in a particular season were pooled together. Such pooling was done for all the analyses to give 46 samples, except for apparent total tract organic matter digestibility (dOM) and ME determination where all the samples of a feed type were pooled together to give one sample per feedstuff, resulting in a total of twelve samples.

### Chemical analyses and *in vitro* incubations

Samples were initially air-dried before transport and then oven-dried at 50°C to constant weight and ground to pass a 1-mm-sieve with a hammer mill (Model MF 10B, IKA Werke, Willmington, NC, USA). Dry matter (DM) concentrations were determined by drying about 0.5 g sample in a forced-air oven at 105°C overnight. Concentrations of crude ash (CA) were determined by incineration at 550°C in a muffle furnace (Model N 11, Nabertherm, Bremen, Germany) and of ether extract (EE) by Soxhlet extraction (Tecator Soxtec System HT 1043 Extraction Unit, Foss Tecator, Eden Prairie, MN, USA). Nitrogen was analysed by Dumas combustion (Vario Max C/N Analyser, Elementar Analysensysteme GmbH, Hanau, Germany) and multiplied by 6.25 to give the CP concentration. Neutral and acid detergent fibre (NDF, ADF) concentrations were determined using an ANKOM^200^ Fibre Analyser (ANKOM Technology, Macedon, NY, USA). Sodium sulphate was used in NDF analysis but without α-amylase [11]. Gross energy (GE) concentrations were determined by bomb calorimetry (C 7000 Isoperibolic, Janke & Kunkel IKA – Analysentechnik, Staufen, Germany). All the analyses were done in duplicate according to VDLUFA [12] and repeated when the standard deviation of the mean of both determinations was greater than 5% of the mean.

Cobalt, molybdenum, and selenium concentrations were determined by Inductively-Coupled Plasma-Mass spectrometry according to method 2.2.2.5 and iron, potassium, sodium, phosphorus, and sulphur concentrations by Inductively-Coupled-Plasma-Optic-Emission spectrometry modified to pressure digestion was used according to method 2.2.2.6 [13].

*In vitro* incubations were done according to Menke and Steingass [14]. Rumen fluid was collected before the morning meal from two rumen-fistulated cows in late lactation fed on a diet made of (per kg; as-fed basis): maize silage (353 g), grass silage (199 g), grass hay (83 g), barley straw (15 g), a concentrate mixture (99 g) mainly composed of barley grain, maize grain, and rapeseed cake, rapeseed extract meal (51 g), and supplement water (200 g). Samples and hay standards of 200 mg were weighed in triplicate into 100 mL calibrated glass syringes. Six additional blank syringes were included in each run. Rumen fluid was mixed with a buffer solution prepared as described in Menke and Steingass [14] immediately before collecting of rumen liquor. Then, 30 mL of the inoculum was dispensed into each syringe and the initial volume recorded. Final volumes of the contents of the syringes were recorded after 24 hours. All samples were incubated for 24 hours, two times each on different days. If relative standard deviation of the results of both days was >5%, the same sample was incubated on a third day. Mean net gas produced during fermentation of the substrate (in mL/200 mg sample DM) was calculated across the two days as the difference between the initial and the final volume of the syringe contents minus the gas production from the blank syringes corrected for day-to-day differences in the gas production from the hay standard. The dOM and ME concentrations were estimated according to Menke and Steingass [14] using the following equations:

$$\begin{array}{l} \text{dOM}=153.8+8.453\text{GP}+0.595\text{CP}+0.675\text{CA};\text{and}\\ \text{ME}=2.2+0.1375\text{GP}+0.0057\text{CP}+0.0002859\text{EE}2; \end{array}$$

where dOM is the apparent total tract organic matter digestibility (g/kg organic matter [OM]), ME is the metabolizable energy concentration (MJ/kg DM), GP is the net gas production after 24 hours of incubation (mL/200 mg DM), CA is the crude ash, CP the crude protein, and EE the ether extract (all in g/kg DM).

Additionally, dOM of the pasture herbage was predicted using two equations from the literature that are based on the chemical composition of tropical grass species derived from *in vitro* estimations using [6]: dOM = 1,395+0.83CP–0.94 NDF–0.74ADF (developed from tropical pasture herbage samples (n = 56) using *in vitro* rumen fluid-pepsin modified Tilley and Terry as reference methodology); and [15]: dOM = 1.22CP+ 47.33 (developed from samples [n = 18] of six tropical grass species using *in vitro* pepsin-cellulase Tilley and Terry as reference methodology); where dOM is the apparent total tract organic matter digestibility (g/kg OM), CP the crude protein, NDF the neutral detergent fiber, and ADF the acid detergent fiber (all in g/kg DM).

### Statistical analyses

Statistical analysis was done using R3.2.5 (R statistical software; R Development Core Team) for descriptive statistics and one-way analysis of variance. The following statistical model was used to analyse the differences in nutritional parameters between the zone and seasons:

$${\text{Y}}_{\text{ij}}=\mu +{\text{S}}_{\text{i}}+{\text{s}}_{\text{j}}+{\text{Ss}}_{\text{ij}}+{ɛ}_{\text{ij}}$$

where Y_ij_ = response parameters; μ = overall mean; S_i_ = effect of the zone, i; s_j_ = effect of the season, j; Ss_ij_ = effect of the interaction between zone and season; and ɛ_ij_ = random effects.

Arithmetic means were compared using multiple comparison tests using Tukey honest significant difference test and differences declared at p<0.05.

## RESULTS

### Nutritive value and biomass yield of pasture herbage

Pasture herbage had the second highest CP concentrations (Table 1) and highest concentrations of phosphorus, sulphur, and molybdenum compared to the other feedstuffs analysed (Table 2). The ME concentrations of the pasture herbage were >7 MJ/kg DM and the dOM was >550 g/kg OM.

**Figure 2:**
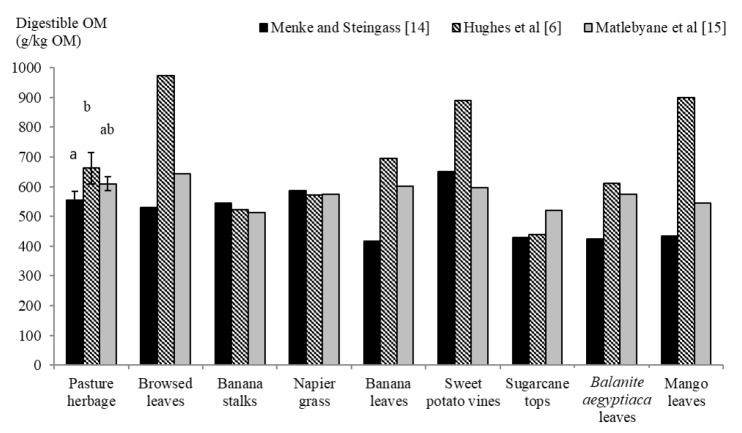
Comparison of apparent total tract organic matter digestibility as estimated from *in vitro* gas production [14] or proximate nutrient and fibre fraction concentrations [6,15] in feedstuffs collected (n = 12 for pasture herbage, and n = 1 each for the rest of the feedstuffs) in Lower Nyando, Western Kenya, during February 2014 and May 2015. OM, organic matter. The error bars represent one standard deviation about the mean. Different letters on error bar imply significant differences (p<0.05).

Methods used to estimate dOM yielded different results (Figure 2) with pronounced differences in both, absolute values and the ranking of feedstuffs according to their dOM.

Seasonal differences were observed for above-ground BY of the pasture herbage, concentrations of DM (p<0.05; Table 3), NDF (p<0.01), ADF (p<0.01), and CP (p<0.001), as well as dOM (p<0.05). Similarly, the concentrations of potassium (p<0.01, Table 4), phosphorus (p<0.001), and sulphur (p<0.05) in pasture herbage differed between seasons with lowest concentrations being observed in the long dry season.

There were significant differences between zones for BY (p< 0.001) and concentrations of CA, NDF, CP, and GE of the pasture herbage (for all parameters p<0.05 except CP, p<0.01, Table 3), with the Highlands having the highest BY (about 2.0 to 2.5 times the BY of the pasture herbage from the other zones) and CP concentrations (i.e., 1.2 to 1.3 times the CP of the pasture herbage from the other zones). Zonal differences were also observed in mineral concentrations for phosphorus (p<0.01, Table 4), potassium and cobalt (p<0.05), and sodium and molybdenum (p<0.001). The pasture herbage in the Highlands had the highest potassium concentrations, whereas that found in Lowlands had the highest phosphorus, cobalt, sodium, and molybdenum concentrations.

### Availability and nutritive value of supplement feedstuffs across zones

The MBL had the highest CP and lowest fibre concentrations compared to other supplementary feedstuffs with the exception of MIL, which had lower NDF concentrations. However, mineral concentrations in MBL were similar to other supplementary feedstuffs. There were fewer supplement feedstuffs on offer in the Mid-slopes and Lowlands than in the Highlands, and they were of poorer nutritive value (3.5 to 8.2 g CP/100 g DM, dOM <430 g/kg OM, and ME<5.9 MJ/kg DM; Table 1) and only available in the long dry season. The concentrations of phosphorus, potassium, iron, and cobalt (Table 2) were highest in supplement feedstuffs in the Highlands. However, feedstuffs in the Mid-slopes had the highest molybdenum concentrations, whereas those of the Lowlands had the highest sodium and sulphur concentrations.

## DISCUSSION

### Nutritive value and biomass yield of pasture herbage

The nutritive value of pasture herbage was higher than of the supplement feedstuffs in the current study and the herbaceous pasture vegetation in Tanzanian rangelands [16]. Mean CP concentration of the pasture herbage was 35% higher than that found in the rangeland vegetation of tropical highlands in Ethiopia [17], and was above the minimum threshold of 70 g/kg DM required for rumen microbial growth and activity. The NDF and ADF concentrations of the pasture herbage were 10% to 31% lower than those reported from East Africa [17,18], whereas phosphorus, sulphur, and molybdenum concentrations of pasture herbage were within the range reported in [16] and about 2 to 8 times higher than those of the supplement feedstuffs analysed in the current study. These mineral concentrations in pasture herbage were adequate for cattle requirements provided that daily feed intake is adequate [19]. Such differences in nutritional value of the herbaceous vegetation on African rangelands could be due to, amongst other factors, differences in climate, soil fertility, species composition, and stage of maturity [20].

Differences in dOM of the feedstuffs in the present study when estimated from *in vitro* gas production and proximate nutrient concentrations or from concentrations of proximate nutrient and fibre fractions could be due to differences in the chemical composition and nutrient degradability of the feedstuffs used to derive the respective equations. For instance, the extraordinarily high dOM estimates from the equation of Hughes et al [6] for feedstuffs with low ADF concentrations (<280 g/kg DM) in the present compared to the pasture herbage may be related to the fact that the equation was developed in herbages rich in ADF (about 422 g/kg DM, standard deviation of 39.7). Values derived from the equation of Matlebyane et al [15] showed small differences in dOM between feedstuffs, possibly because CP, which was the only independent variable of the equation, may not contribute much on its own to the overall dOM of the analysed feedstuffs. Although both equations based on concentrations of proximate nutrients or fibre fractions were derived for tropical ruminant feedstuffs, neither of them was developed based on *in vivo* data. The *in vitro* gas production equation proposed by Menke and Steingass [14] to estimate dOM of feedstuffs, has been derived from *in vivo* data of a broad range of feedstuffs which, although not tropical, covered the range of nutritive value of the pasture herbage reported here (n = 185; *in vivo* dOM range of 293 to 800 g/kg OM). Hence, although accuracy of the dOM and ME estimates cannot be quantified here, because respective *in vivo* data is lacking, those derived from *in vitro* gas production appear to be more robust. Nevertheless, results imply that there is a need to validate or develop new equations based on *in vivo* data for estimating dOM and ME of tropical ruminant feedstuffs. Mean dOM and ME concentrations derived from *in vitro* gas production of 554 g/kg OM and 7.1 MJ/kg DM were comparable to some cultivated temperate grass hays [20], and even higher than those of the Napier grass analysed in the current study, supporting the assertion that the pasture herbage was of moderate to good nutritive value. The relatively low nutritive value of Napier grass in the present study may be due to the fact that farmers in the study region tend to harvest plants at a very mature stage to achieve higher BY.

### Temporal differences in biomass yield and nutritive value of pasture herbage

Seasonal differences in BY, concentrations of DM, NDF, ADF, CP, and dOM were observed for the pasture vegetation, which may be related to differences in plant growth rates and stage of plant maturity. It is important to note that there were only minor differences in precipitation (CV, 3% to 17%) and ambient temperatures (CV, 3% to 4%) between seasons (Figure 1) with the exception of the rainfall in long dry (driest period, 96 to 117 mm/month) and the long wet seasons (wettest period, 141 to 186 mm per month) for which also the most pronounced differences in vegetation parameters were found. Across all zones, the BY was highest in the long wet season (i.e., 1.3 to 3.0 times higher than in other seasons). However, surprisingly, concentrations of NDF and ADF were highest and CP concentrations and dOM lowest in the long wet season. That may have been, at least partly, due to rapid growth and accumulation of biomass, aided by high rainfall at the beginning of the long wet season, which was not consumed by the animals due to use of enclosure, resulting in lower value herbage at harvest during mid-season.

Seasonal changes in mineral concentrations of the herbaceous vegetation of native tropical pastures are related to a translocation of minerals to seeds or the root system and/or a dilution process during plant growth with advancing plant maturity [21]. An adult dry non-pregnant cow in Lower Nyando has a mean liveweight of 206 kg with a mean daily gain of approximately 50 g/d. The daily ME requirements for maintenance and liveweight gain of such an animal would be approximately 35 MJ [22]. Given the ME concentrations of the pasture herbage in the long wet and long dry seasons (Table 3), cows would need to consume 5.4 kg DM/d and 4.9 kg DM/d of pasture during the long wet and long dry season, respectively, to meet these requirements. The DM intake would provide approximately 16 g/d and 8 g/d of phosphorus in the long wet and long dry seasons, respectively, based on the mean phosphorus concentrations in the pasture vegetation of 0.29 g and 0.17 g/100 g DM in both seasons (Table 4). This would exceed the daily phosphorus requirements defined by NRC [19] of 10 g/d phosphorus in the long wet season, but is below the recommendations during the long dry season. In contrast to previous reports of mineral deficiencies in the Rift Valley of Kenya [5], concentrations of other macro- and micro-minerals seem sufficient to meet the requirements defined by NRC [19] of cattle at moderate to low performance levels even during the long dry season. Such evaluations based on mineral concentrations do not take into account that not all of the minerals contained in the feedstuffs are bioavailable and further studies should analyse the bioavailability of minerals from pasture herbage in tropical grasslands to evaluate its potential contribution to meeting the animals’ mineral requirements. Nevertheless, results suggest a need for supplemental feeding in particular in the long dry season to prevent mineral deficiencies which may considerably reduce animal health and performance.

### Spatial differences in biomass yield and nutritive value of pasture herbage

Across the four seasons, the differences between zones in BY and concentrations of CA, CP, and NDF of the pasture vegetation were likely due to differences in rainfall and ambient temperature and livestock husbandry (e.g., in the Highlands cattle graze in paddocks, while in the Lowlands they are tethered or herded). For instance, the Highlands are characterized by the highest rainfall of the three zones (Figure 1), promoting plant growth and BY on pastures and likely increasing leaf:stem ratios in plant biomass associated with higher CP concentrations in total above-ground plant biomass [23], which may explain the higher CP concentrations in samples of the pasture vegetation in the Highlands in the current study. The N contents in the soils are 2.1 times higher in the Highlands than in the Lowlands and 1.2 times higher than in the Mid-slopes [24]. Along with the higher BY, the higher CP concentrations indicate that carrying capacity of the pastures in the Highlands may be greater than of those in the other two zones.

Pasture herbage in the Lowlands had the highest concentrations of phosphorus, sodium, iron, cobalt, and molybdenum. In contrast, the herbage in the Mid-slopes had the lowest concentrations of phosphorus, potassium, sodium, and cobalt. Site differences could possibly be due to erosion of soils with minerals in particular in the Mid-slopes leading to deposition in the Lowlands. Another reason for difference in mineral concentrations between zones may be the fact that the clay soils in the Lowlands are poorly drained. Water logging in the Lowlands may limit the availability of some minerals such as potassium whose uptake in water logged soils may be inhibited by a decrease in root cell energy caused by oxygen deficiency within the soil pore spaces [25]. Irrespective of the zonal differences in mineral concentrations, with the exception of phosphorus and sodium, mean concentrations of all minerals in the pasture herbage across all seasons were within the range or above those recommended by NRC [19] for diets of cattle.

### Availability and nutritive value of supplement feedstuffs in the zones

The common supplement found in all the zones is MBL that was rich in CP likely due to the inclusion of leaves of leguminous shrub and tree species such as *Acacia* spp., *Sesbania* spp., and *Calliandra* spp. in plant samples. Additionally, ADF and NDF concentrations of MBL were lower and thus dOM higher than values determined in previous studies [18]. The mineral concentrations in MBL were also higher than most of the other supplement feedstuffs analysed in the current study or published for browse leaves in the literature. For instance, the concentrations of phosphorus were marginally higher than those determined by Dickhoefer et al [26] for leaves and twigs of native shrubs and trees in semi-arid, sub-tropical highland regions of Oman. The CP and selenium concentrations in MBL were much higher than in pasture herbage across all the zones and seasons, while both had about the same dOM and ME concentrations. Assuming there were no limiting effects of anti-nutritional factors, MBL can thus be used as CP and selenium supplement to pasture herbage in all the zones. Nevertheless, further studies should be carried out on the anti-nutritional factors in MBL so as to evaluate its suitability as a supplement feedstuff.

The main supplement feedstuffs used in the Mid-slopes and the Lowlands during the long dry season, when pasture herbage is scarce, are sugarcane tops and purchased rice husks and straw. Additionally, BAL, and as a last resort, MIL are fed to ruminant livestock in the Lowlands. The availability of BAL and MIL is limited; thus, for instance, the use of MIL to supplement selenium which it has in high concentrations may not be feasible. The use of sugarcane tops and rice straw as nutrient supplement to grazing cattle is limited by their low dOM values. Hence, feed and feeding management strategies such as a physical, chemical, or biological treatment of crop residues or the strategic supplementation with purchased concentrate feedstuffs might be viable options for livestock farmers in these systems to increase feed intake and nutrient supply in domestic ruminant livestock during the dry season.

In the Highlands, a broader range of supplement feedstuffs was available. Feedstuffs such as maize thinnings are only occasionally used and thus of less relevance [27]. Banana leaves and pseudostem and Napier grass are available all year round as supplement feedstuffs and commonly fed to dairy cattle. The CP, NDF, and ADF concentrations of Napier grass were similar to reported values [28]. Despite lower CP concentrations, Napier grass makes a good supplement in addition to grazing of pastures given that its dOM and ME values were higher than those of the pasture herbage. Additionally, Napier grass had higher concentrations of cobalt and selenium. Napier grass nutritive value could however, be even further improved by identifying optimum cutting frequency and height, and increased manure application [28]. Additionally, SPV is abundant in the Highlands at the beginning of the long dry season following its harvest after the short cropping season. The CP concentration of SPV was higher and the NDF and ADF concentrations were lower than in most supplement feedstuffs analysed in the present study, resulting in highest dOM amongst all the feedstuffs. The leaf BY of SPV has been reported to range between 0.9 t to as much as 2.8 t DM/ha in different agro-ecological zones of Kenya [29]. Moreover, the higher concentrations of CP and cobalt in SPV compared to the pasture vegetation imply that, if properly managed and conserved, SPV can be used as CP and cobalt supplement in addition to grazing the native pastures, particularly during the long dry season.

The high potassium concentrations in the supplement feedstuffs are consistent with reports of potassium abundance in other tropical feedstuffs [30], as is the case of low sodium concentrations in tropical forages due to low sodium levels in tropical soils. Generally, the iron and selenium concentrations were higher than those previously reported from East Africa [30]. The existing supplement feedstuffs in all zones had lower concentrations of phosphorus, sodium, sulphur, and molybdenum compared to the pasture vegetation. Hence, they cannot compensate for the phosphorus and sodium deficiencies noted in pasture vegetation.

The observed differences in BY and nutritive value of the pasture vegetation between zones, and the local availability of supplement feedstuffs need zone-specific feeding strategies. The Highlands are more suitable for dairy farming than the other two zones due to high BY of the herbaceous pasture vegetation and the better nutritive value of the supplement feedstuffs. There is however, a potential for intensification in the Mid-slopes and the Lowlands, for example by increasing the variety of feed resources, improving forage husbandry, and processing of crop residues.

## CONCLUSION

In Western Kenya, pasture herbage is of superior nutritive value than commonly available supplement feedstuffs. The highland regions are more suited to animal production due to higher herbaceous BY on native pastures and greater diversity of available supplement feedstuffs. There is need for supplemental feeding in the long dry season and locally available feedstuffs may at least partially compensate for nutritional deficiencies in the pasture vegetation. However, together with the lack of valid approaches to estimate dOM and ME of tropical ruminant feedstuffs, the spatial and temporal variability in the nutritional value of feedstuffs for domestic ruminants shows need for considerable safety margins in diet formulation and for region- and season-specific solutions to improve animal nutrition and performance.

## Figures and Tables

**Table 1:** Nutrient concentrations, organic matter digestibility, and metabolizable energy concentrations of common feedstuffs fed to ruminants in Lower Nyando, Western Kenya, between February 2014 and May 2015 (Arithmetic mean±one standard deviation)

Zone	Feedstuff	n	n*	DMg/kg FM	CA	NDF	ADF	CP	EE	dOM1)g/kg OM	GE	ME1)
	
					------------------------------- g/kg DM -----------------------------						------ MJ/kg DM ------	
Highlands	Napier grass	8	2	195±34.8	168±39.4	653±19.5	376±14.1	83±13.1	7.0	587	14.5±0.21	7.0
	Banana pseudostem	10	2	85±58.2	111±24.4	655±49.3	382±56.4	32±6.6	8.0	544	15.3±0.36	7.1
	SPV	3	2	259±109.0	85±11.2	407±31.1	278±13.6	101±11.0	19.0	650	16.8±0.09	8.9
	Banana leaves	5	1	142±26.0	159±7.5	562±10.1	350±19.6	105±18.0	45.0	416	17.2	4.3
	Maize thinnings	1	1	301	137	685	372	95	16.0	576	15.1	7.1
Mid-slopes	Sugarcane tops	4	3	642±373.9	52±4.7	741±29.4	395±21.4	38±6.1	6.0	430	17.2±0.46	5.9
	Swamp reeds	2	1	460±321.0	56±7.2	714±27.5	353±14.2	41±7.2	6.0	430	15.6	5.9
Lowlands	BAL	2	1	475±118.4	66±7.4	594±13.3	396±12.7	82±8.6	8.0	425	18.5	5.5
	Rice stover	1	1	912	159	640	365	46	nd	nd	nd	nd
	Rice husks	1	1	845	45	709	371	35	nd	nd	nd	nd
	MIL	1	1	475	152	367	273	60	24.0	435	16.0	4.8
All zones	Pasture herbage	75	12	328±174.1	104±16.4	626±33.6	321±30.3	111±25.7	12.0±1.90	554±30.0	16.6±0.35	7.1±0.42
	MBL	22	6	377±118.6	69±22.7	371±39.5	255±27.2	140±25.9	22.0	530	18.8±0.56	7.0

n, original number of samples; n*, number of pooled samples (samples collected in the same zone and during the same season were pooled to give one pool sample for analysis); DM, dry matter; FM, fresh matter; CA, crude ash; NDF, neutral detergent fibre; ADF, acid detergent fibre; CP, crude protein; EE, ether extract; dOM, apparent total tract organic matter digestibility; OM, organic matter; GE, gross energy; ME, metabolizable energy; SPV, sweet potato vines; BAL, *Balanite aegyptiaca* leaves; nd, not determined; MIL, *Mangifera indica* leaves; MBL, mixed browsed leaves.

1) As estimated from *in vitro* gas production and proximate nutrient concentrations using equations of Menke and Steingass [14].

**Table 2:** Mineral concentrations of common feedstuffs fed to ruminants collected on native pastures in Lower Nyando, Western Kenya, between February 2014 and May 2015 (Arithmetic mean±one standard deviation)

Zone	Feedstuff	n	n*	P	K	Na	S	Fe	Co	Mo	Se
	
				------------------------- g/kg DM -----------------------				---------------------- mg/kg DM ------------------------			
Highlands	Napier grass	8	2	1.5±0.61	42.3±5.09	0.03±0.01	1.1±0.32	1,588±1,796	0.4±0.38	0.9±0.53	0.2±0.01
	Banana pseudostem	10	2	0.6±0.19	54.2±4.89	0.03±0.00	0.4±0.06	622±240	0.2±0.13	1.0±0.50	0.1±0.05
	SPV	3	2	2.2±0.99	34.1±2.40	0.04±0.00	1.5±0.26	1,142±1,030	0.4±0.08	0.4±0.31	0.1±0.01
	Banana leaves	5	1	1.3	24.3	0.01	1.5	375	0.1	0.7	0.2
	Maize thinnings	1	1	1.8	19.1	0.03	1.2	1,825	0.7	0.4	0.1
Mid-slopes	Sugarcane tops	4	3	0.9±0.17	12.3±5.32	0.02±0.02	0.7±0.13	144±50	0.1±0.04	0.8±0.72	0.1±0.01
	Swamp reeds	2	1	1.0	11.1	0.05	1.0	282	0.1	1.8	0.1
Lowlands	BAL	2	1	0.8	17.1	0.11	2.0	255	0.1	0.1	0.2
	Rice stover	1	1	1.3	23.4	0.47	1.0	531	0.6	0.9	0.1
	Rice husks	1	1	0.9	15.9	0.02	0.8	149	0.04	0.3	0.1
	MIL	1	1	0.8	7.0	0.06	1.3	136	0.1	1.1	0.5
All zones	Pasture herbage	75	12	2.9±0.85	26.5±6.41	0.10±0.07	2.2±0.40	769±362	0.3±0.12	2.9±1.43	0.1±0.03
	MBL	22	6	1.8±0.37	20.0±2.28	0.03±0.02	1.9±0.44	297±255	0.2±0.11	0.9±0.45	0.2±0.35

n, original number of samples; n*, number of pooled samples (samples collected in the same zone and during the same season were pooled to give one pool sample for analysis); P, phosphorus; K, potassium; Na, sodium; S, sulphur; DM, dry matter; Fe, iron; Co, cobalt; Mo, molybdenum; Se, selenium; SPV, sweet potato vines; BAL, *Balanite aegyptiaca* leaves; MIL, *Mangifera indica* leaves; MBL, mixed browsed leaves.

**Table 3:** Nutritional value of the above-ground herbaceous biomass on native pastures in Lower Nyando, Western Kenya, as determined for different zones and seasons during August 2014 to May 2015 (Arithmetic mean±one standard deviation)

Season/zone	n	BY t DM/ha	DM g/kg FM	CA	NDF	ADF	CP	EE	dOM1)g/kg OM	GE	ME1)
	
				-------------------------------- g/kg DM -------------------------------						---------- MJ/kg DM -------	
Season											
Short dry	4	2.0^ab^±1.03	204^a^±61.4	106^a^±9.9	614^ab^±15.4	308^a^±13.1	123^a^±13.1	14^a^±1.8	572^ab^±8.9	16.5^a^±0.23	7.3^a^±0.23
Short wet	4	3.4^bc^±2.99	350^ab^±125.9	107^a^±12.2	608^a^±25.0	303^a^±16.1	130^a^±21.8	13^a^±1.8	581^a^±28.4	16.4^a^±0.34	7.4^a^±0.53
Long dry	4	1.4^a^±0.65	546^b^±154.5	95^a^±23.6	634^ab^±44.2	315^a^±29.7	98^b^±21.1	10^a^±1.6	540^ab^±21.6	16.8^a^±0.37	7.0^a^±0.17
Long wet	4	4.3^cd^±1.61	211^a^±42.0	108^a^±15.6	647^b^±31.1	360^b^±20.2	95^b^±27.6	12^a^±1.7	523^b^±15.7	16.5^a^±0.46	6.7^a^±0.27
SEM		0.57	72.3	6.9	9.0	9.2	4.1	1.0	4.8	0.19	0.23
p2)		0.002	0.011	0.286	0.009	0.002	<0.001	0.077	0.024	0.227	0.057
Zone											
Lowlands	3	1.9^a^±1.20	315^a^±189.0	113^a^±15.7	615^a^±32.9	321^a^±30.2	103^a^±22.8	13^a^±1.4	565^a^±23.2	16.3^a^±0.27	7.2^a^±0.35
Mid-slopes	3	2.3^a^±1.24	363^a^±184.3	92^b^±11.9	639^b^±34.3	328^a^±35.2	109^a^±21.5	12^a^±2.5	557^a^±41.3	16.9^b^±0.22	7.3^a^±0.51
Highlands	3	4.7^b^±2.74	314^a^±144.9	102^ab^±11.3	632^ab^±30.3	316^a^±26.6	129^b^±27.2	11^a^±0.7	540^a^±24.4	16.5^ab^±0.32	6.8^a^±0.34
SEM		0.52	62.6	5.9	7.8	7.9	3.6	0.9	12.8	0.17	0.20
p2)		<0.001	0.687	0.025	0.024	0.259	0.001	0.054	0.230	0.035	0.156

n, number of pooled samples (samples collected in the same zone and during the same season were pooled to give one pool sample for analysis); BY, above-ground biomass yield of the pasture herbage; DM, dry matter; FM, fresh matter; CA, crude ash; NDF, neutral detergent fibre; ADF, acid detergent fibre; CP, crude protein; EE, ether extract; dOM, apparent total tract organic matter digestibility; OM, organic matter; GE, gross energy; ME, metabolizable energy; SEM, standard error of mean.

1) As estimated from *in vitro* gas production and proximate nutrient concentrations using equations of Menke and Steingass [14].

2) Season×zone interactions were not significant.

a–d Superscripts in the same column with different letters denote significant differences between seasons or zones (p<0.05).

**Table 4:** Mineral concentrations of herbaceous vegetation collected on native pastures in Lower Nyando, Western Kenya, as determined for different zones and seasons during August 2014 to May 2015 (Arithmetic mean±one standard deviation)

Season/zone	n	P	K	Na	S	Fe	Co	Mo	Se
	
		--------------------------------- g/kg DM ---------------------------				-------------------------------- mg/kg DM -------------------------			
Season									
Short dry	4	3.5^a^±0.49	28.8^a^±3.46	0.1^a^±0.08	2.5^a^±0.07	802^a^±198	0.3^a^±0.04	3.1^a^±1.57	0.1^a^±0.01
Short wet	4	3.5^a^±0.31	28.6^a^±4.36	0.1^a^±0.07	2.6^a^±0.26	877^a^±597	0.3^a^±0.16	3.1^a^±1.77	0.1^a^±0.01
Long dry	4	1.7^b^±0.30	17.8^b^±1.56	0.1^a^±0.11	1.7^b^±0.06	987^a^±174	0.4^a^±0.17	2.1^a^±0.86	0.1^a^±0.02
Long wet	4	2.9^ab^±0.70	30.9^a^±5.93	0.1^a^±0.06	2.1^ab^±0.40	410^a^±106	0.2^a^±0.10	3.1^a^±1.96	0.1^a^±0.06
SEM		0.15	1.81	0.02	0.19	235	0.07	0.40	0.03
p1)		<0.001	0.002	0.935	0.021	0.191	0.190	0.148	0.736
Zone									
Lowlands	3	3.3^a^±0.86	25.7^ab^±6.15	0.2^a^±0.03	2.2^a^±0.29	950^a^±453	0.4^a^±0.12	4.6^a^±1.08	0.1^a^±0.05
Mid-slopes	3	2.4^b^±0.78	23.4^b^±4.72	0.1^b^±0.01	2.1^a^±0.53	550^a^±278	0.2^b^±0.09	2.4^ab^±0.21	0.1^a^±0.02
Highlands	3	3.0^a^±0.86	30.5^a^±7.45	0.1^b^±0.02	2.3^a^±0.46	807^a^±298	0.2^ab^±0.06	1.7^b^±0.27	0.1^a^±0.01
SEM		0.13	1.57	0.02	0.17	203	0.06	0.35	0.02
p1)		0.002	0.013	<0.001	0.538	0.244	0.034	<0.001	0.480

n, number of pooled samples (samples collected in same zone and during the same season were pooled to give one pool sample for analysis); P, phosphorus; K, potassium; Na, sodium; S, sulphur; DM, dry matter; Fe, iron; Co, cobalt; Mo, molybdenum; Se, selenium; SEM, standard error of mean.

1) Season×zone interactions were not significant.

a,b Superscripts in the same column with different letters denote significant differences between seasons or zones (p<0.05).
